# Noble Metal-Based Catalysts with Core-Shell Structure for Oxygen Reduction Reaction: Progress and Prospective

**DOI:** 10.3390/nano12142480

**Published:** 2022-07-19

**Authors:** Chao Wang, Cuihua An, Chunling Qin, Hassanien Gomaa, Qibo Deng, Shuai Wu, Ning Hu

**Affiliations:** 1 Key Laboratory of Hebei Province on Scale-Span Intelligent Equipment Technology, School of Mechanical Engineering, School of Materials Science and Engineering, Hebei University of Technology, Tianjin 300401, China; wangchao19972021@163.com (C.W.); ancuihua@hebut.edu.cn (C.A.); clqin@hebut.edu.cn (C.Q.); 2 Department of Chemistry, Faculty of Science, Al-Azhar University, Assiut 71524, Egypt; h.gomaa@azhar.edu.eg; 3 State Key Laboratory of Reliability and Intelligence Electrical Equipment, Hebei University of Technology, Tianjin 300130, China; ninghu@hebut.edu.cn; 4 National Engineering Research Center for Technological Innovation Method and Tool, School of Mechanical Engineering, Hebei University of Technology, Tianjin 300401, China

**Keywords:** core–shell nanostructure, oxygen reduction reaction, electrocatalyst, noble metal-based

## Abstract

With the deterioration of the ecological environment and the depletion of fossil energy, fuel cells, representing a new generation of clean energy, have received widespread attention. This review summarized recent progress in noble metal-based core–shell catalysts for oxygen reduction reactions (ORRs) in proton exchange membrane fuel cells (PEMFCs). The novel testing methods, performance evaluation parameters and research methods of ORR were briefly introduced. The effects of the preparation method, temperature, kinds of doping elements and the number of shell layers on the ORR performances of noble metal-based core–shell catalysts were highlighted. The difficulties of mass production and the high cost of noble metal-based core–shell nanostructured ORR catalysts were also summarized. Thus, in order to promote the commercialization of noble metal-based core–shell catalysts, research directions and prospects on the further development of high performance ORR catalysts with simple synthesis and low cost are presented.

## 1. Introduction

With the development and progress of science and technology, the exploitation and utilization of fossil fuels has reached its peak [1,2,3,4,5,6,7,8,9]. However, fossil fuel is a kind of non-renewable energy. Due to continuous exploitation, the content of fossil fuels in the earth is decreasing. Moreover, the generated gas after the combustion of fossil fuels has caused serious pollution to the environment, resulting in a large number of natural disasters. Therefore, the development of a new type of sustainable clean energy and efficient utilization system is particularly important to solve the current problems such as environmental pollution and energy shortages [10,11,12,13,14,15,16,17,18,19,20]. Different from the traditional internal combustion engine, which converts the chemical energy of fuel molecules into mechanical energy via combustion, a fuel cell is a device that converts the chemical energy of fuel molecules directly into electric energy through an electrochemical reaction. It is considered as one of the candidates for the next generation of energy because of its high energy conversion efficiency, its environmentally friendly characteristics, high energy density and so on [21,22]. However, the oxygen reduction reaction (ORR) at the cathode has become the biggest limiting factor for proton exchange membrane fuel cells (PEMFCs). Due to its slow kinetics, the application of fuel cells depends on a large number of platinum (Pt) catalysts. Due to the low reserves, Pt is extremely expensive, which means cathode catalysts account for about half of the commercial production cost of fuel cells. Therefore, reducing the loading of Pt and improving the efficiency of catalysts represent a very important research area in the field of fuel cells [23,24,25].

The core–shell nanomaterials are composed by a shell (outer material) and core (inner material). The core–shell structural noble metal-based nanomaterials are generated when a noble metal is used as the shell. Among many ORR catalysts, core–shell structural noble metal-based nanomaterials are more advanced. Core–shell structural noble metal-based catalysts have the following two main advantages: on the one hand, it uses the relatively cheap core material as the carrier and to support the noble metal material in the outer layer, which is able to greatly reduce the preparation cost of the catalyst. On the other hand, the synergy effect of the core and shell can improve the ORR activity and stability of the catalyst in a complex reaction environment. The synergy effect is mainly determined by the following three kinds of effects: (1) the ligand effect: the different composition of core and shell leads to electron transfer and the change of band structure; (2) the strain effect: the tension or compression of the noble metal shell lattice causes the change of surface adsorption energy; (3) the geometric effect: the different three-dimensional structure of surface atoms can affect the electrochemical properties. Through the combination of these effects and theoretical calculation, core–shell structural noble metal-based nanomaterials with better ORR properties can be reasonably designed and prepared [26,27,28].

In recent years, a large number of core–shell structural noble metal-based ORR catalyst with ultra-high activity have been successfully designed and synthesized. The attention has gradually shifted to the economic benefits such as saving costs and simplifying the preparation process. So, efforts to create a simpler and batch-operable preparation method which can decrease the dosage of noble metals are underway. For example, the traditional preparation method of the underpotential deposition (UPD) method requires a potentiostat to accurately control the reaction potential. Additionally, the reaction environment is harsh which is not suitable for batch production [29,30]. A synthesis strategy of the spontaneous deposition of copper without electrochemical equipment, reductants and stabilizers was designed [31]. The above strategy not only greatly reduces the requirement of reaction conditions, but also improves the ORR performances of the catalysts. In order to reduce the consumption of noble metals, a series of investigations were carried out on the thickness of the shell. Additionally, costs can be reduced by decreasing the thickness of the shell. A series of highly efficient catalysts with only monolayer shells were obtained, which exhibited ultra-high stability [32,33]. The ORR performances of core–shell structural noble metal-based catalysts can be affected by many factors. Hence, in order to design core–shell structural noble metal-based catalysts that are more suitable for commercial production, more information about the factors affecting ORR performance needs to be elucidated.

This work summarizes a large number of research achievements in recent years from four aspects: synthesis methods, temperature, kinds of doping elements and the number of shell layers. The elements influencing the ORR performances and regulation strategies of core–shell structural noble metal-based catalysts are reviewed according to the view of commercial production. A testing technology which is beneficial to conversion from half-cell testing to practical application is introduced. This work provides effective guidance for the preparation procedure, noble metal dosage and ORR performance optimization of core–shell structural noble metal-based catalysts. It also promotes the commercial production of core–shell structural noble metal-based catalysts and promotes their practical application in fuel cells.

## 2. ORR Testing Technology

The ORR process involves multiple intermediates and multi-step reactions. Many factors, such as electrode potential, type of catalyst, crystal plane structure, reaction temperature and so on, could affect the corresponding electrochemical performances [34,35,36]. The lack of experimental verification methods for the reaction process results in accurate ORR mechanisms not being obtained [37]. However, in recent years, researchers have begun to describe the ORR mechanism by means of characterization [38,39,40] and theoretical calculation [41], which is a benefit in the design of catalysts that meet the requirements. After the desired structural catalysts are obtained, the ORR activity and durability of the catalysts can be analyzed by measuring the electrochemically active surface area (ECSA), Tafel slope, the onset potential (E_onset_) and the half-wave potential (E_1/2_) [42,43,44,45,46]. Two testing techniques are described in detail below.

### 2.1. Testing Technology of Thin-Film Rotating Disk Electrode (TF-RDE)

**Figure 1:**
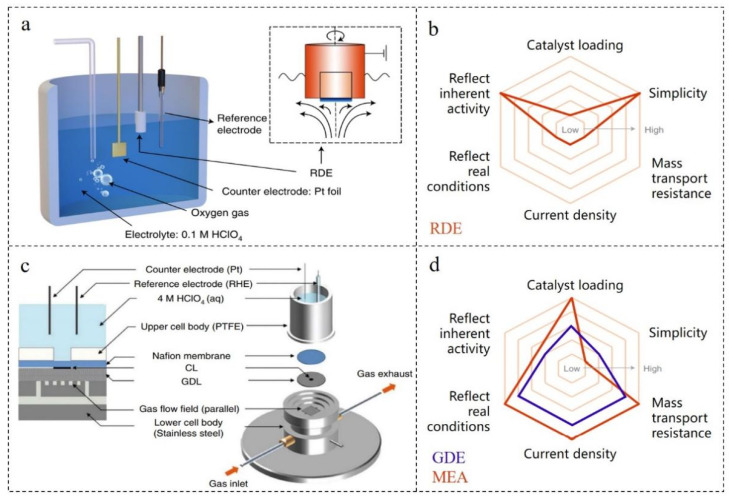
(**a**) The RDE configuration. (**b**) Radar charts of RDE. (**c**) The GDE configuration. (**d**) Radar charts of GDE and MEA. Reproduced with permission from [54]. Copyright © 2022, Nature.

In principle, the newly synthesized ORR catalyst should be tested and evaluated in the real working environment of the fuel cell, but this testing method is often not feasible in practice. On the one hand, the preparation of membrane electrode assembly (MEA) requires professional skills, complex expansion equipment and a large number of catalysts, so it is difficult to prepare a good MEA in the general laboratory. On the other hand, a rapid screening technique for performance is needed in the early stage of catalyst preparation. However, the complexity of MEA preparation cannot achieve the purpose of rapid screening. TF-RDE is a common laboratory technology based on commercial RDE technology (Figure 1a), which can test the ORR performance of milligram catalysts [47]. TF-RDE is supported by complete hydrodynamic equations and convection–diffusion equations. In the process of continuous improvement, it provides a fine method for membrane preparation technology and electrochemical parameter control [48,49]. Because of its simple and fast operation, TF-RDE is widely used in the laboratory. Additionally, there is a standard operation flow that can compare the performances of different catalysts to a certain extent [50,51]. However, it should be noted that there are still differences in the details of the experimental schemes and methods of TF-RDE, which may lead to different measured catalyst activities or different results.

### 2.2. Testing Technology of Gas Diffusion Electrode (GDE)

Although TF-RDE is practical for the initial screening of catalysts in the laboratory, some shortcomings of TF-RDE limit its ability to predict catalysts in complex battery environments. On the one hand, in the procedure of TF-RDE, the reaction gas (H_2_, O_2_ or air) is charged into the electrolyte. However, the low solubility of the gas in the electrolyte is very different from the gas concentration in practical application [52]. On the other hand, the catalyst layer used in TF-RDE is usually less than 1 μm, while the thickness is greater than 5 μm in actual fuel cells. In addition, the catalyst coverage area is also very different. So, there is a great difference in the catalyst activity measured by TF-RDE and MEA [53]. Therefore, GDE testing technology has been proposed, which is an intermediate technology between TF-RDE and MEA (Figure 1b–d) [54]. Through the improvement and supplement of GDE testing technology, the gap between laboratory catalyst testing and actual fuel cell catalyst testing can be narrowed under simple and fast conditions [55,56,57].

GDE is a kind of electrode with a high active surface area and high catalytic activity according to the principle of fuel cells. The principle is to keep the gas and electrolyte in equilibrium in the micropores at atmospheric pressure. Then, the potential is controlled to cause an electrochemical reaction while a stable gas–liquid–solid three-phase interface is formed [58]. At present, GDE testing can check the relevant catalyst layer structure, current and potential range similarly to MEA testing technology. Additionally, it can ensure the comparability, speed and accuracy of half-cell experiments. Therefore, GDE test is considered to be an important step from TF-RDE test to MEA test in the prediction of catalyst application performance.

## 3. Factors Affecting the ORR Performances of the Core-Shell Structural Noble Metal-Based Nanomaterials

### 3.1. Synthesis Method

In the ORR reaction, the composition, structure and morphology of the catalyst are important elements that impact electrochemical performance. The synthesis strategy determines the morphology, structure and composition of the catalyst. Therefore, in order to obtain high ORR stability and activity, the synthesis strategy should be adjusted to regulate the performance of the catalyst. In the following, several synthesis strategies of core–shell structural noble metal-based catalysts are briefly introduced and their ORR performances are compared.

#### 3.1.1. Electrodeposition Method

**Figure 2:**
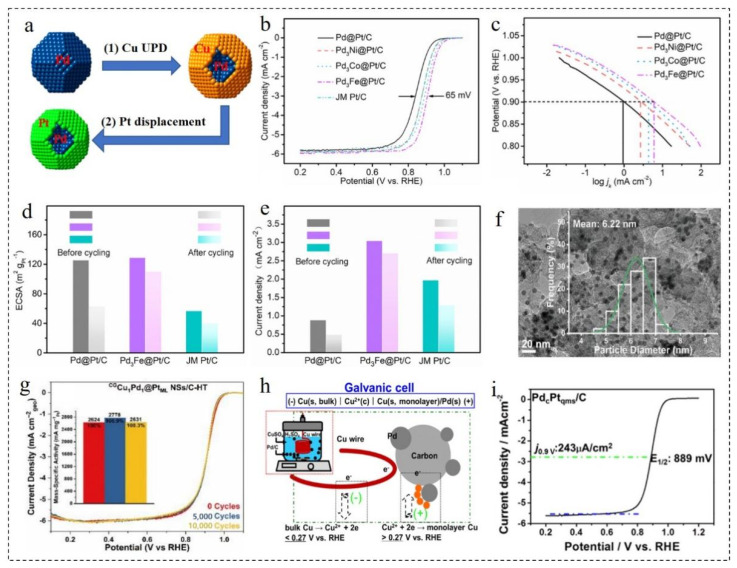
(**a**) Schematic Illustration of UPD process with copper as sacrificial layer. Reproduced with permission from [61]. Copyright © 2022, American Chemical Society. (**b**) LSV curves (10 mV·s^−1^, 1600 rpm) and (**c**) Tafel plots of Pd_3_M@Pt/C NPs in O_2_-saturated 0.1 M HClO_4_. (**d**) ECSA and (**e**) ORR current density at 0.9 V (vs. RHE) of Pd_3_Fe@Pt/C NPs at the before and after 10,000 cycles. Reproduced with permission from [62]. Copyright © 2022, Elsevier. (**f**) Representative TEM image and the associated particle diameter distribution histogram of ^CG^CuPd@Pt/C NPs. (**g**) LSV curves (10 mV·s^−1^) of ^CG^Cu_1_Pd_1_@Pt in O_2_-saturated 0.1 M HClO_4_ solutions before and after the multiple cycles. Reproduced with permission from [63]. Copyright © 2022, Royal Society of Chemistry. (**h**) Principle and experiment of Pd@Pt/C growth. (**i**) LSV curves (10 mV·s^−1^, 1600 rpm) of Pd@Pt/C from (**h**) in O_2_-saturated 0.1 M HClO_4_. Reproduced with permission from [31]. Copyright © 2022, American Chemical Society.

Electrodeposition has become a common method for the synthesis of metal elements, but it is not easy to control the thickness and quantity of shells. Therefore, UPD is widely used as a more “intelligent” surface-limited electrodeposition method [59]. UPD is an excellent method for the preparation of core–shell structures, especially for the preparation of core–shell nanomaterials with ultra-thin shells or even monolayer shells. The UPD method means that when the equilibrium potential of a metal is slightly positive, the metal can be deposited on the surface of the core using the electrochemical method. Additionally, the metal deposited on the surface of the core is then replaced with the desired shell metal to form a metal shell [60]. Figure 2a schematically illustrates the UPD process with Cu as the sacrificial layer. Due to the underpotential, the Cu layer reduces the deposition rate of Pt and promotes the surface diffusion of Pt adsorbed atoms, and a smooth Pt monolayer is formed on the surface of Pd [61]. Pd_3_M/C (M=Fe, Ni and Co) nanoparticles (NPs) with 20 wt% metal loading were prepared using a two-step method [62]. Then, Pt was deposited on the surface of Pd_3_M using the UPD method to obtain Pd_3_M@Pt/C NPs with a monolayer Pt shell. As shown in Figure 2b,c, the ORR activity increased significantly with the addition of the transition metal (Fe, Co or Ni). Among them, Pd_3_Fe@Pt/C NPs exhibited the best ORR activity, and the mass activity (1.14 A·mg_Pt_^−1^) was 5.4 times higher than that of commercial Pt/C. It was confirmed that the ORR activity greatly improved by the monolayer uniform Pt shell obtained via the UPD method. In addition, the stability and durability of the catalyst were greatly improved after the addition of Fe in the core. After 10,000 cycles, the ECSA and ORR activity of Pd_3_Fe@Pt/C decreased by 14.5% and 11.1%, respectively. It was much less than the decline in the ECSA and ORR activity of Pd@Pt/C (Figure 2d,e).

Spherical CuPd NPs with a composition-graded (CG) structure were synthesized using the solvothermal method [63]. ^CG^CuPd/C was obtained using simple heat treatment after loading on carbon black. Then, ^CG^CuPd@Cu/C NPs covered by monolayer Cu were obtained by UPD of Cu. Finally, the monolayer Cu was replaced by the galvanic substitution of Pt^2+^, and ^CG^CuPd@Pt/C NPs with monolayer Pt were obtained. As shown in Figure 2f, ^CG^CuPd@Pt/C NPs had a highly uniform, small size and well-defined spherical morphology. The associated particle diameter distribution histogram demonstrates that the average diameter of NPs was 6.22 nm, and the standard deviation was 7.2%. The ^CG^CuPd@Pt/C catalyst showed better ORR activity in acid electrolyte. The specific activity, noble metal mass activity and Pt mass activity of the catalyst in acid ORR were 1.110 mA·cm^−2^, 680 mA·mg^−1^ and 2624 mA·mg^−1^_Pt_, respectively, which were 3.4, 3.4 and 13.3 times higher than those of commercial Pt/C. In addition, after 10,000 cycles, the changes in size, morphology and distribution were negligible compared with the initial structure. As displayed in Figure 2g, the mass activity of ^CG^CuPd@Pt/C slightly increased at first and then decreased in the long cycle experiment. However, the final mass activity was still higher than the initial value, which further proved that they had excellent stability.

Loichet Torres et al. [64] developed a method to promote the UPD of Cu on Pt surfaces in a hydrogen (H_2_) atmosphere. First of all, the amount of Cu deposited in the presence of dissolved H_2_ was determined by rapid stripping voltammetry. Then, the air-saturated Cu^2+^ electrolyte was exposed to gas containing H_2_, and the open-circuit potential drop of the Pt disk and Pt/C film electrode was monitored simultaneously. The results showed that when the gas ratio was 0.1% and 3% of the H_2_/Ar mixture, the Pt@Cu NPs covered with a monolayer shell could be obtained. Additionally, Pt@Cu_UPD_/C catalysts of gram scale could be obtained through the improvement of the electrolytic cell. Inspired by the primary battery, Wang et al. [31] designed a method for the synthesis of Pd@Pt/C catalysts, which required neither electrochemical equipment nor reductants and stabilizers. As shown in Figure 2h, when Pd NPs came into contact with Cu wire in a solution containing Cu^2+^, Cu spontaneously deposited on the surface of Pd under the condition of an open circuit and a monolayer Cu shell was formed. Then, the monolayer Pt shell was obtained using a simple displacement reaction to form Pd@Pt/C NPs with the desired structure. As observed during the electrochemical test, the E_1/2_ of the catalyst was 0.889 V, which was much better than the catalyst prepared using the traditional method (Figure 2i). Moreover, the preparation process was simple, and did not require electrochemical equipment and special control, and had a good prospect of mass production.

#### 3.1.2. Chemical Reduction Method

The chemical reduction method is widely used in the preparation of core–shell structural noble metal-based materials. The effects of the reaction solvent, end-capping agent and reducing agent on the structure and ORR performances of the catalysts have been studied systematically [65]. Chemical reduction methods are generally classified as a continuous reduction method or co-reduction method. The continuous reduction method, also known as the seed growth method, first reduces a metal salt to a “crystal seed” (the core of the NPs) in water or organic solvents. Then, another metal is deposited on the core through the seed growth process to form a core–shell structure. Another method is co-reduction method, also known as simultaneous reduction method. When the reaction occurs, there are two kinds of metal salt precursors at the same time. The metal with high redox potential is first reduced to form the core. Additionally, the metal with a low oxidation-reduction potential is reduced and deposited on the core to form the core–shell structure [66].

HAuCl_4_·4H_2_O is first added into oleamine; then, Au NPs are reduced by vigorous stirring at a high temperature (150 °C) [67]. Finally, Ni(acac)_2_ is added for the reduction of Ni^2+^. In the reaction process, Au NPs not only seed but also catalyze the reduction of Ni^2+^. Ni atoms continue to grow on the Au core to form a shell of about three atomic layers. Then, Pd(acac)_2_ is added to obtain Au@NiPd NPs through a further reduction reaction. In addition, a strategy for the synthesis of core–shell materials using the co-reduction method was designed [68]. Pd(acac)_2_, HAuC1_4_·4H_2_O and Fe(acac)_3_ were used as metal precursors and heated in oleamine containing oleic acid. A series of Au@FePd NPs with different Fe-Pt ratios were directly obtained after one-pot reactions.

**Figure 3:**
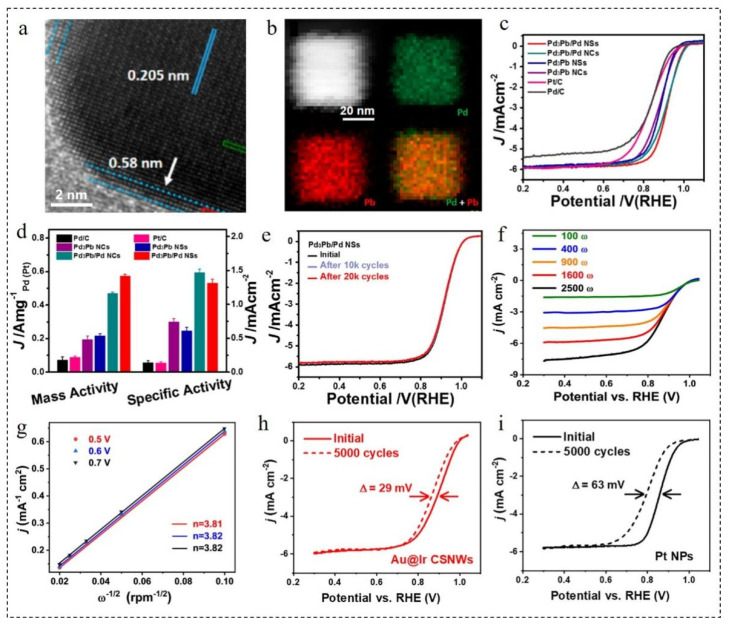
(**a**) HRTEM image, (**b**) HAADF-STEM image and corresponding elemental mappings of Pd_3_Pb@Pd nanosheets. (**c**) LSV curves and (**d**) mass and specific activities of Pd_3_Pb@Pd nanosheets in O_2_-saturated 0.1 M KOH solution. (**e**) LSV curves of Pd_3_Pb@Pd nanosheets before and after different potential cycles between 0.6 and 1.1 V (vs. RHE). Reproduced with permission from [69]. Copyright © 2022, American Chemical Society. (**f**) LSV curves of Au@Ir nanowires in O_2_-saturated 0.1 M KOH solution at different rotation rates. (**g**) Koutecky–Levich plot of Au@Ir nanowires towards ORR at 0.6 V potential. LSV curves (5 mV·s^−1^, 1600 rpm) of (**h**) Au@Ir NPs and (**i**) Pt NPs in O_2_-saturated 0.1 M KOH solution before and after the multiple cycles. Reproduced with permission from [70]. Copyright © 2022, Elsevier.

Tang et al. [69] synthesized Pd_3_Pb@Pd nanosheets using Pd(acac)_2_ and Pb(HCOO)_2_ as metal precursors, oleylamine/octadecene mixture as the solvent and surfactant, glucose as the reductant and NH_4_Br as the structure guiding agent. The resulting material had uniform tensile strain at the top and edge (Figure 3a,b). As shown in Figure 3c–e, the ORR mass activity and specific activity of Pd_3_Pb@Pd at 0.9 V were 0.57 A·mg_Pd_^−1^ and 1.31 mA·cm^−2^, which were 6.5 and 9.8 times that of commercial Pt/C, respectively. The activity of Pd_3_Pb@Pd nanosheets with a uniform Pd shell decreased and the composition almost remained the same after 20,000 cycles. An one-pot chemical reduction strategy was developed for the rapid synthesis of high quality Au@Ir nanowires in 1-naphthol ethanol solution [70]. HAuCl_4_ and IrCl_3_·H_2_O were added into the mixed solution of water and ethanol. Then, a 1-naphthol ethanol solution was added and soaked at 75 °C for 12 h to obtain Au@Ir nanowires. As displayed in Figure 3f,g, at 0.5 V, 0.6 V and 0.7 V, the transferred electron numbers of ORR in Au@Ir nanowires were 3.81, 3.82 and 3.82, respectively. It indicated that a desirable 4e^−^ path occurred at Au@Ir nanowires. The obtained materials not only possessed good ORR activity and methanol tolerance, but also had remarkable stability. After 5000 cycles, the E_1/2_ of Au@Ir nanowires degraded to 29 mV, while the E_1/2_ of the commercial Pt NPs (achieved from Johnson Matthey Corporation) degraded to 63 mV (Figure 3h,i).

#### 3.1.3. Other Methods

The chemical reduction and electrochemical methods mentioned above are commonly used in the preparation of core–shell structural noble metal-based materials [33]. In addition, there are many other preparation methods that can synthesize ORR catalysts with excellent structure and performance. Among them, dealloying is not only a high-quality method for the preparation of porous materials, but also widely used in the preparation of solid core–shell catalysts. Porous materials with a large number of pores lead to a rapid decline in stability and ORR activity. On the other hand, the solid core–shell catalyst has strong core and shell protection, which can provide better durability and performance [71].

**Figure 4:**
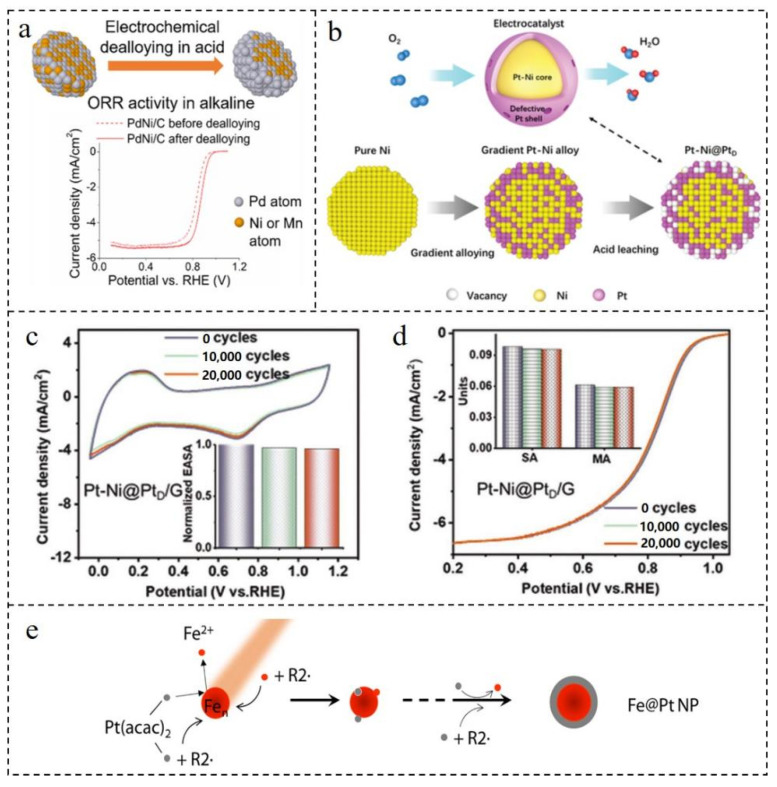
(**a**) Schematic diagram of PtNi@Pt/C principle and LSV curves of ORR before and after de-alloying. Reproduced with permission from [74]. Copyright © 2022, American Chemical Society. (**b**) Design schematic diagram of the “defective Pt-skin armored” PtNi@Pt_D_ catalyst. (**c**) CV and (**d**) LSV of PtNi@Pt_D_ catalyst before, after 10,000 and 20,000 potential cycles between 0 and 1.1 V (vs. RHE) in O_2_-saturated 0.1 M HClO_4_ at a scan rate of 50 mV·s^−1^. Reproduced with permission from [75]. Copyright © 2022, Wiley-VCH. (**e**) Mechanism of single-step synthesis of Fe@Pt NPs by sonochemistry. Reproduced with permission from [77]. Copyright © 2022, Elsevier.

The deposition of a Pt monolayer on Au substrates is a powerful strategy used to maximize the use of Pt atoms. However, the Pt deposited on Au substrates provides far from a smooth monolayer, and rather produces a rough three-dimensional nanocluster [72]. In order to solve the above problem, a synthesis strategy was explored so that a smooth Pt monolayer could grow on the surface of an Au core, in which the Au core was obtained by dealloying CuAu alloy [73]. On the one hand, using dealloyed CuAu NPs as the core could reduce the dosage of noble metal Au. On the other hand, it can be used as a catalyst to promote the growth of the Pt monolayer on the surface of Au, making it better resemble a smooth monolayer. A type of Pd-Ni@Pd/C NPs was synthesized by means of electrochemical dealloying [74]. Because the 3D transition metal Ni formed an alloy phase with Pd and the lattice constant was less than Pd, PdNi NPs were selected as the representative to study the dealloying effect. In 0.1 M HClO_4_ solution, the non-noble metal Ni was selectively leached across 10 cycles between 0.05 V and 1.1 V (RHE) to form core–shell structural PdNi@Pd/C NPs with rich Pd on the surface, as shown in Figure 4a. Compared with the unalloyed PdNi/C, the E_1/2_ of PdNi@Pd/C increased by 36 mV, and the specific activity of Pd/C was 3.3 times higher than that of Pd/C at 0.85 V. After 16,000 cycles, the E_1/2_ of PdNi@Pd/C NPs only decreased 14 mV. The galvanic substitution reaction was used to achieve the gradient distribution of Pt-Ni composition from surface to interior [75]. Additionally, the “defective-armored” structure of Pt shell and Pt-Ni core NPs decorated on graphene (PtNi@Pt_D_/G) were obtained by etching the superficial Ni atoms. The Pt shell had a large number of defects, and the principle and structure diagram are displayed in Figure 4b. The Pt skeleton of the shell could not only protect the core from being dissolved, but also adjusted the electronic structure of the shell which benefits to increase the active sites. After testing, the ORR mass activity of the PtNi@Pt_D_/G catalyst was 3 times higher than commercial Pt/C. The ECSA could still maintain 96% after 20,000 cycles, and the mass activity was stable at 0.059 A·mg_Pt_^−1^ (Figure 4c,d). Compared with Pt, Ni was better able to dissolve and reduce the stability of the catalyst under long-term operation. However, the PtNi@Pt_D_/G catalyst had excellent stability, which proved that the strong Pt shell prevented the dissolution of Ni in the core. A Pd@Pt/C catalyst was prepared using a direct displacement reaction [76]. The specific method was to directly replace Pd NPs with PtCl_4_^2−^ in an N_2_-saturated H_2_SO_4_ solution at 70 °C to form a uniform Pt shell-wrapped Pd@Pt/C catalyst. The whole chemical reaction is as follows (Equation (1)):

(1) $$\mathrm{Pd}/C+K_{2}{\mathrm{PtCl}}_{4}=\mathrm{Pt}@\mathrm{Pd}/C+K_{2}{\mathrm{PdCl}}_{4}$$

Compared with the potential difference between Cu and PtCl_4_^2−^ in UPD, the smaller potential difference between the Pd core and PtCl_4_^2−^ slowed down the reaction rate and was more beneficial to the formation of a uniform Pt shell. The obtained Pt@Pd/C catalyst showed good ORR performance, and the mass activity (at 1.0 mA·cm^−2^) was 2.4 times higher than that of commercial Pt/C. The direct displacement reaction made it easier to prepare catalysts which was expected to achieve mass production in the future. In order to optimize the synthesis steps, ORR catalysts with excellent performance were synthesized using a simpler method. Park et al. [77] reported a one-step synthesis of Fe@Pt NPs via the sonochemical method, also known as ultrasound-assisted polyol synthesis (UPS). Through detailed analysis and careful observation of the reaction process, a fine mechanism for the formation of Fe@Pt NPs using a UPS reaction was proposed (Figure 4e). The proposal of the reaction mechanism would play an important guiding role in controlling the reaction and promote the industrial application of core–shell catalysts synthesized via the UPS method.

### 3.2. Effect of Temperature on ORR Performances

**Figure 5:**
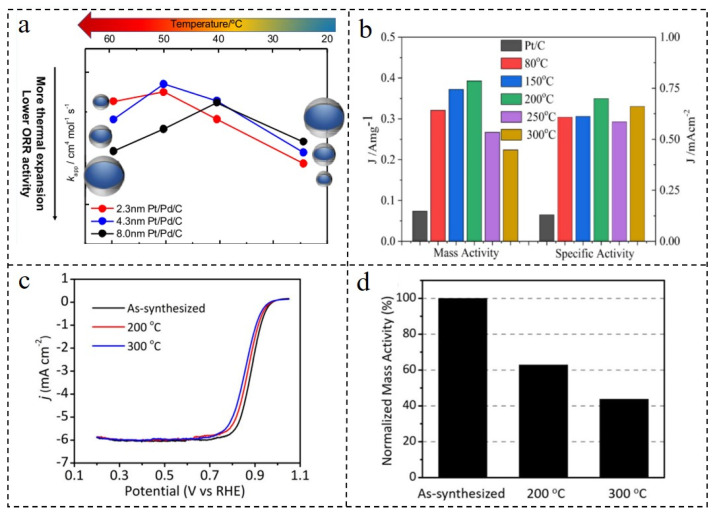
(**a**) Regularity of ORR activity of Pd core with different sizes changing with temperature. Reproduced with permission from [82]. Copyright © 2022, American Chemical Society. (**b**) Mass activity and specific activity at 0.9 V_RHE_ of Pd@PtNi/C catalyst at different annealing temperatures histogram. Reproduced with permission from [83]. Copyright © 2022, Elsevier. Comparison of LSV curves (**c**) and Pt mass activity at 0.9 V_RHE_ (**d**) of as-synthesized and annealed Pd@Pt/C. Reproduced with permission from [84]. Copyright © 2022, Elsevier.

In recent years, the specific reason why the activity evaluation of the same catalyst in MEA is often lower than that of RDE has not been accurately explained [78,79]. In the actual fuel cell working environment and RDE test environment, temperature is a crucial factor affecting the performance of the catalyst. So, researchers have studied the influence of temperature on the structure and performance of the catalyst [80]. The study of the relationship between temperature and ORR performance would help to correctly select the working environment of the catalyst. It is extremely significant to improve the efficiency of a catalyst in practical application [81]. The ORR activity of Pd@Pt/C NPs with different sizes is different between 25 °C and 60 °C [82]. Figure 5a shows that the ORR activity of the Pd@Pt/C catalyst varied with temperature. The apparent rate constant of the Pd@Pt/C catalyst was higher than that of commercial Pt/C at 25 °C, but began to decrease at 50–60 °C. The smaller Pd@Pt/C NPs had better ORR activity at 25 °C, but the ORR activity began to decrease when the temperature reached 60 °C. The oxide coverage of Pd@Pt/C NPs was much lower than that of commercial Pt/C at 25 °C. The oxide coverage increased sharply with the increase in temperature, which was probably the reason for the decrease in ORR activity mentioned above.

The preparation of a core–shell catalyst also requires reasonable control of the reaction temperature. A reaction temperature that is too low cannot guarantee effective reduction which leads to the shelling being shell incomplete, while a higher temperature causes element segregation and leads to an uneven shell composition. Therefore, the reasonable selection of the reaction temperature during the preparation of the catalyst can effectively improve the ORR activity of the catalyst. Pd@Pt-Ni/C catalysts were prepared using an efficient and simple method [83]. Firstly, Pd@Pt-Ni/C intermediates were prepared using an effective solvothermal method. Then, the core–shell structural Pd@Pt-Ni/C catalysts were prepared by heat treatment in an H_2_ atmosphere at 150, 200, 250, 300 °C for 3 h, respectively. As shown in Figure 5b, according to the results, it could be seen that the mass activity and specific activity of Pd@Pt-Ni/C catalysts treated at 200 °C were the highest, and they were 5.2 and 5.3 times higher than the commercial Pt/C, respectively. The E_1/2_ also reached its maximum at 200 °C (0.88 V), which was much higher than for Pt/C (0.82 V). In addition, the results of transmission electron microscopy (TEM) and electrochemical measurements were also consistent. The shell on the surface of NPs heat-treated at 200 °C was uniform and complete. The surface structure and ORR activity of Pd@Pt NPs also changed after heat treatment at different temperatures [84]. The Pt mass activity of the Pd@Pt/C catalyst at 0.9 V decreased by 37% when annealing to 200 °C and by 56% after annealing at 300 °C (Figure 5c,d). According to the TEM images, it could be seen that the decrease in activity was due to the fact that the annealing treatment destroyed the core–shell structure of Pd@Pt/C, so that Pt atoms could not be fully utilized. The mixing of Pd and Pt atoms occurred at 200 °C. Additionally, the ORR activity of the catalyst decreased significantly in the range of 200–300 °C. The above study provides guidance and predictions for the dispersion technology of mass heat production of catalysts and provides a theoretical basis for the assembly and manufacture technology of membrane electrodes which requires hot pressing.

### 3.3. Influence of Strain Effect

**Figure 6:**
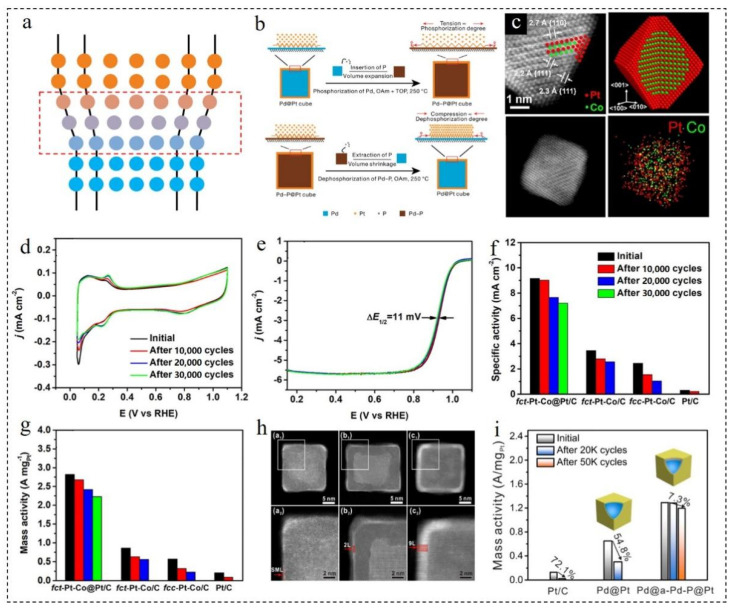
(**a**) Schematic of the strain field influenced by lattice mismatch at the interface. Reproduced with permission from [85]. Copyright © 2022, Nature. (**b**) A method for controlling lattice strain of ultrathin Pt shells. Reproduced with permission from [88]. Copyright © 2022, Nature. (**c**) Schematic, HAADF-STEM images and EDX mapping of PtCo@Pt NPs. (**d**) CV and (**e**) LSV curves recorded from the fct-PtCo@Pt/C NPs before and after different cycles. (**f**) Specific and (**g**) mass activity of the catalysts at 0.9 V (vs. RHE) before and after different cycles. Reproduced with permission from [89]. Copyright © 2022, American Chemical Society. (**h**) HAADF-STEM images and (**i**) ORR performances of Pd@a-Pd-P@Pt. Reproduced with permission from [95]. Copyright © 2022, American Chemical Society.

When nanomaterials are composed of two components with different crystal structures or lattice parameters, the lattice mismatch results in an interaction between the two components. The strain effect is the phenomenon that eventually leads to corresponding changes in the atomic and electronic structures. In the process of the formation of the core–shell material, the strain effect occurs when the lattice parameters of the shell and the core material do not match. This leads to a certain degree of tension or compression of the shell material. It is worth noting, however, that this effect requires a certain range of shell thickness (Figure 6a). The tension of the shell lattice enhances the adsorption of the reaction intermediates, because the d-band center moves up relative to the Fermi level. On the contrary, compression weakens the adsorption of reaction intermediates. The strain effect changes the surface adsorption capacity of the catalyst and further changes the performance of the catalysts [85].

#### 3.3.1. Effect of Core Doping

Shell strain can be regulated by controlling the core element doping of core–shell NPs, which is a common method by which to improve catalytic efficiency [86,87]. Inserting atoms into the core to increase the core volume leads to the expansion of the shell lattice, while the extraction of doped atoms from the core reduces the core volume and shrinks the shell lattice. He et al. [88] firstly deposited Pt on a Pd cube to form Pd@Pt NPs, and then inserted the P element into the Pd core to expand the Pt shell lattice, or the Pt shell was deposited onto the Pd-P core, and then the P was removed to obtain a compressed Pt shell, which is shown in Figure 6b. Shell strains of −5.1% and 5.9% were obtained by phosphating and dephosphorization, which adjusted the catalytic performance of the catalyst to a great extent.

In addition, doped atoms affect the performance and structure of catalysts because of their own properties. The difficulty of controlling the arrangement of shape and surface atoms at the same time was overcome [89]. In their work, they synthesized a type of PtCo@Pt octahedral catalyst. The PtCo@Pt catalyst had a face-centered tetragonal core and an ultra-thin Pt shell with (111) crystal plane orientation. As displayed in Figure 6c, from the high angle annular dark field scanning transmission electron microscopy (HAADF-STEM) image, it can be seen that the core atomic layers of Pt and Co appeared alternately and were highly ordered. The shell was composed of about 3–4 Pt atomic layers with the dominance of (111) facets on the surface. The ORR mass activity (2.82 A·mg^−1^) and specific activity (9.16 mA·cm^−2^) of the NPs were 13.4 and 29.5 times higher than those of commercial Pt/C, respectively. The excellent ORR activity was mainly due to two factors. The first is the surface lattice strain caused by the ordered insertion of Co atoms in the core. Secondly, the arrangement of the Pt surface of the shell maximized the exposure of the surface active sites. As shown in Figure 6d–g, there is a correlation between the mass activity and specific activity decrease in fcc-PtCo/C and fct-PtCo/C NPs. It indicates that the decline in the catalyst performance in the long cycle is related to the leaching of Co and the loss of surface active sites. The core–shell structural noble metal-based NPs with doping 3D transition metals are easy to dissolve and agglomerate in the harsh working environment of the actual fuel cell, which greatly reduces the efficiency and durability of the catalyst. Therefore, a new phosphating method based on the thermal decomposition of NaH_2_PO_2_ at 250–300 °C was designed [90]. The PtP_1.4_@Pt/C catalyst was obtained by acid etching, and the excellent ORR performances were measured. The mass activity and specific activity of the catalyst at 0.9 V (vs. RHE) were 0.31 mA·μg_Pt_^−1^ and 0.62 mA·cm^−2^, which were 2.1 and 2.8 times higher than those of commercial Pt/C, respectively. More importantly, after 30,000 cycles, the mass activity of PtP_1.4_@Pt/C NPs decreased by only 6%, while that of commercial Pt/C decreased by 46%. The high stability of PtP_1.4_@Pt/C NPs was mainly due to the electronic structure effect caused by P doping. Due to the electronic structure effect caused by P doping, the PtP_1.4_@Pt/C NPs had high durability. Besides, P doping increased the anchoring effect of the carbon carrier on catalyst particles and prevented their migration and agglomeration.

In order to fix the core without it being dissolved, the traditional design strategy is to thicken the shell to protect the core. However, this greatly reduces the ECSA and mass activity, which is not conducive to reducing the cost [91,92]. Therefore, in order to improve the durability and stability of the core–shell structure, the protection of the shell cannot be completely relied upon and inherently stable core materials need to be identified or synthesized [93,94]. Pd@a-Pd-P@Pt catalysts were obtained by depositing Pt atoms on an amorphous palladium phosphide (a-Pd-P) substrate [95]. Additionally, the thickness of the Pt layer could be flexibly controlled between the sub-monolayer and ninth layer. As shown in Figure 6h,i, the Pt shell was uniformly wrapped around the cube, and the substrate shape and shell uniformity were not vulnerable to destruction when adjusting the shell thickness. Among them, Pd@a-Pd-P@Pt_SML_ with a sub-monolayer Pt shell had the best ORR performance. The ORR mass activity of Pd@a-Pd-P@Pt_SML_ was as high as 4.08 A·mg_Pt_^−1^ and 1.37 A·mg_Pt+Pd_^−1^ at 0.9 V (vs. RHE). After 50,000 cycles, the activity loss was only 9%, and the structural deformation was negligible. The amorphous Pd-P formed by doping P in the core of Pd had high corrosion resistance, which could reduce the required shell thickness of the catalyst and optimize the utilization of Pt, while maintaining ultra-high durability. This work expanded on the application of amorphous materials in the field of core–shell catalysts.

#### 3.3.2. Effect of Shell Doping

The doping of noble-metal shells has become a commonly used method to control the ORR performance of catalysts. Doping does not only reduce the dosage of noble metals, but also introduce strain to change the surface electronic structure. When the diameter of the doped atom is smaller than that of the shell, compressive strain is introduced into the shell. Otherwise, tensile strain is introduced. In addition, the stability and anti-poisoning performance of the catalyst can be improved according to the properties of the doped elements [96,97].

**Figure 7:**
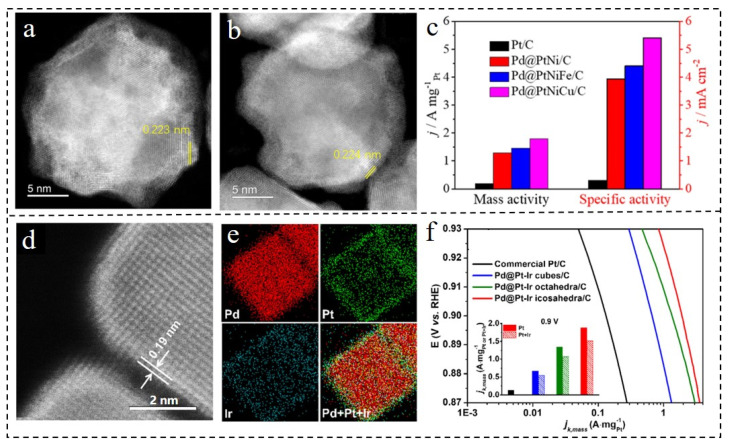
HR-STEM images of (**a**) Pd@PtNiFe NPs and (**b**) Pd@PtNiCu NPs. (**c**) Mass activity and specific activity at 0.9 V_RHE_ of (**a**). Reproduced with permission from [98]. Copyright © 2022, Elsevier. (**d**) Atomic-resolution HAADF-STEM images and (**e**) EDX mapping of Pd@PtIr NPs. (**f**) Mass activities of the Pd@PtIr/C catalysts. Reproduced with permission from [99]. Copyright © 2022, Elsevier.

Based on the principle of the core–shell structure and alloy structure, Zhang et al. [98] successfully synthesized Pd@PtNiFe and Pd@PtNiCu catalysts by doping other metal elements into the shell. In the XRD pattern of the prepared catalyst, it was found that the diffraction peak of Pt had an obvious positive shift relative to Pt (PDF#65-2868), indicating that the alloy insertion of the shell was effective. As seen from the energy dispersive spectrometer (EDS) images in Figure 7a–c, the prepared catalyst had a typical core–shell structure. Besides, Pt, Ni and Fe (or Cu) were uniformly distributed around the Pd core. The ORR activity significantly improved after adjusting the shell composition. In the N_2_-filled environment of the 0.1 M HClO_4_ solution, the Pd@PtNi/C catalyst had an ORR mass activity of 1.29 A mg_Pt_^−1^ compared with Pd@Pt/C catalysts. When the third metal was doped into the shell to form Pd@PtNiFe and Pd@PtNiCu NPs, the ORR mass activity of the catalyst was 1.1 and 1.4 times higher than that of the Pd@Pt/C catalyst. Additionally, the activity declined by only 11.0% and 10.6% after 5000 cycles. A class of highly efficient ORR catalysts with shell doping of Ir was reported [99]. Pd@PtIr NPs in the geometric shapes of cubes, octahedrons and icosahedrons were obtained by regulation. Due to the difference in atomic number, there was an obvious brightness contrast between the core Pd and the PtIr shell in the HAADF-STEM image. A thin PtIr shell around the core Pd was observed, and the EDS image shows that the Ir was highly dispersed in the Pt shell (Figure 7d,e). The average atomic layers of the shells were quantitatively analyzed by inductively coupled plasma mass spectrometry (ICP-MS). The shell thicknesses of the three nanocrystals were all between 1.5 and 1.7 atomic layers. In the N_2_ environment of the 0.1 M HClO_4_ solution, the J_k,specific_ of Pd@PtIr cubes/C, octahedra/C and icosahedra/C was 2.6, 4.8 and 5.8 times higher than that of commercial Pt/C (0.22 mA·cm^−2^). At the same time, J_k,mass_ was 5.2, 10.3 and 14.5 times higher than that of commercial Pt/C (0.13 A·mg_Pt_^−1^) (Figure 7f). Ir is a thermodynamically stable element which is resistant to surface leaching and segregation in an acidic environment [100,101]. The doping of Ir in the Pt shell reasonably regulates the strain effect of the shell, which can effectively improve the ORR catalytic activity while ensuring its stability and durability. This opens the door for the reasonable design of such structures to improve the service life of the catalyst.

**Figure 8:**
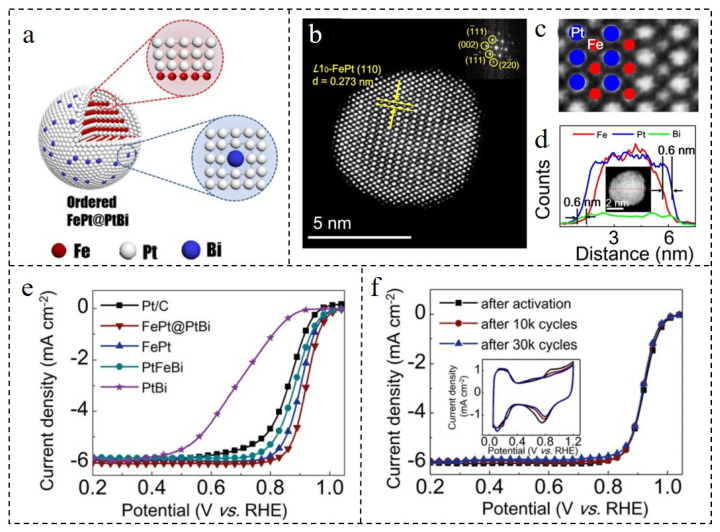
(**a**) The structure diagram of FePt@PtBi. HAADF-STEM images of (**b**) FePt@PtBi and (**c**) FePt core. (**d**) EDS line-scanning of FePt@PtBi. (**e**) LSV curves (5 mV·s^−1^) of FePt@PtBi, FePt, PtFeBi, PtBi and Pt/C in O_2_-saturated 0.1 M HClO_4_ solution. (**f**) LSV curves (100 mV·s^−1^) of FePt@PtBi in N_2_-saturated 0.1 M HClO_4_ solution before and after the multiple cycles, and the corresponding CV curves. Reproduced with permission from [102]. Copyright © 2022, Wiley-VCH.

A series of highly efficient core–shell structural catalysts can be obtained by coating PtBi on the surface of Pt or Pt alloy [102]. A structure diagram of FePt@PtBi NPs with the best ORR performance is provided in Figure 8a. The FePt@PtBi NPs consist of an FePt core and a uniform PtBi shell of 2–3 layers (Figure 8b–d). The mass activity (0.96 mg_Pt_^−1^) and specific activity (2.06 mA·cm^−2^) of FePt@PtBi NPs were 7 and 11 times higher than those of commercial Pt/C, respectively. The E_1/2_ (0.921 V) of FePt@PtBi NPs was higher than the three electrocatalysts (FePt: 0.902 V; PtFeBi: 0.882 V; commercial Pt/C: 0.870 V). Additionally, the activity decreased by only 18% after 30,000 cycles (Figure 8e,f). Compared with the traditional Pt-based electrocatalyst, FePt@PtBi had better anti-poisoning ability against methanol and carbon monoxide. The efficient catalytic activity was attributed to the compressive strain of the core FePt. Besides, the in-plane shear caused by the large amount of Bi in the shell was another favorable factor. The synergistic effect of these two factors could increase the ORR activity of the catalyst. Bi has the ability to increase the resistance to CH_3_OH and CO poisoning [103], so FePt@PtBi NPs also has a strong anti-poisoning ability. This design strategy effectively solved the problem of low tolerance in that high efficiency ORR catalysts are susceptible to CO and CH_3_OH poisoning [104].

### 3.4. Effect of Shell Layer Number on the Performance of ORR

In the core–shell structure, the strain effect caused by lattice mismatch is usually more obvious around the sixth layer of the shell, while the ligand effect plays a leading role in the first 1–2 layers of the shell [105]. In most cases, the ligand effect and the strain effect are synergistic and additive in terms of adsorption energy, but some studies have found that the strain effect is contrary to the ligand effect. Therefore, the selection of an appropriate shell thickness is particularly important in designing ORR catalysts.

**Figure 9:**
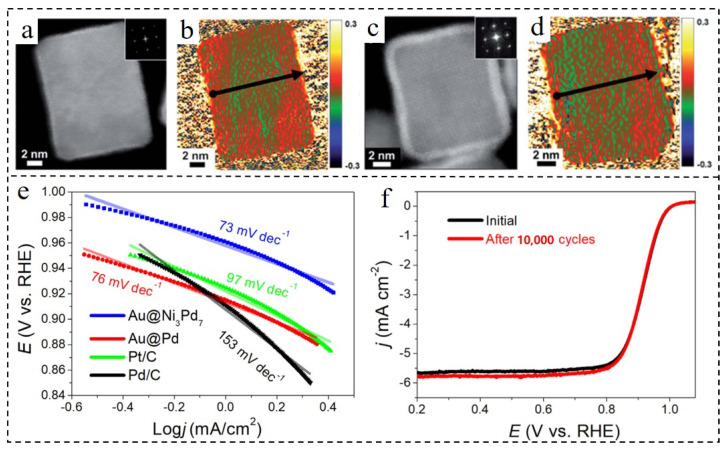
HAADF-STEM images and corresponding GPA for the 2–4 ML (**a**,**b**) and 5–7 ML (**c**,**d**) shelled Pd@Pt nanocubes. Reproduced with permission from [32]. Copyright © 2022, Royal Society of Chemistry. (**e**) Tafel plots over Au@Ni_3_Pd_7_ NPs, Au@Pd NPs, commercial Pd/C catalyst, and commercial Pt/C catalyst in 0.1 M KOH. (**f**) ORR polarization curves of Au@Ni_3_Pd_7_ NPs before and after 10,000 cycles. Reproduced with permission from [67]. Copyright © 2022, Elsevier.

In order to explore the relationship between shell layer number and strain effect, Gamler et al. [32] synthesized Pd@Pt, Rh@Pd and Rh@Pt bimetallic nanocubes with different shell layer numbers. Samples with 2–4 shell layers and 5–7 shell layers were successfully prepared and are displayed in Figure 9a–d, respectively. Red (warm color) represents the relative expansion region and green (cool color) indicates the relative compression region. The high concentration of red area is mainly distributed in the outer layer (Figure 9a,b). However, the thickness of the red region was narrower than that of the shell, which indicates that lattice expansion was more obvious in the outermost layer. Compared with the samples with 5–7 shell layers, the thickness of the outer red area of the samples with 2–4 shell layers was significantly higher. Combined with characterization and theoretical calculation, it was found that the surface of the material with fewer shell layers had larger compressive strain. In Pd@Pt, Rh@Pd and Rh@Pt materials, the compressive strain decreased gradually after about 5–6 ML. Zeng et al. [67] modified the preparation method of Au@Ni NPs and synthesized Au@NiPd catalyst with a shell of only three atomic layers by galvanically substituting Pd^2+^ and shell Ni. With the decrease in shell layers, the tensile strain caused by lattice mismatch between Au with larger lattice parameters and Pd with smaller lattice parameters became more obvious. This changed the electronic structure of the Pd shell, and the d-band center moved upward compared with the unstretched shell. It can be seen from Figure 9e that the Tafel slope of Au@NiPd NPs was only 73 mV·dec^−1^, indicating that the Au@NiPd NPs had the fastest ORR kinetics compared with the other three materials. When the atomic ratio of Ni to Pd was 3/7, the E_1/2_ of Au@NiPd NPs in the alkaline medium was 0.92 V, while the specific activity was 3.7 mA cm^−2^, and the mass activity at 0.9 V was 0.65 A·mg^−1^. As shown in Figure 9f, after 10,000 cycles, Au@NiPd NPs almost retained their original structure and little change occurred in E_1/2_, which was improved compared to that of commercial Pt/C.

**Figure 10:**
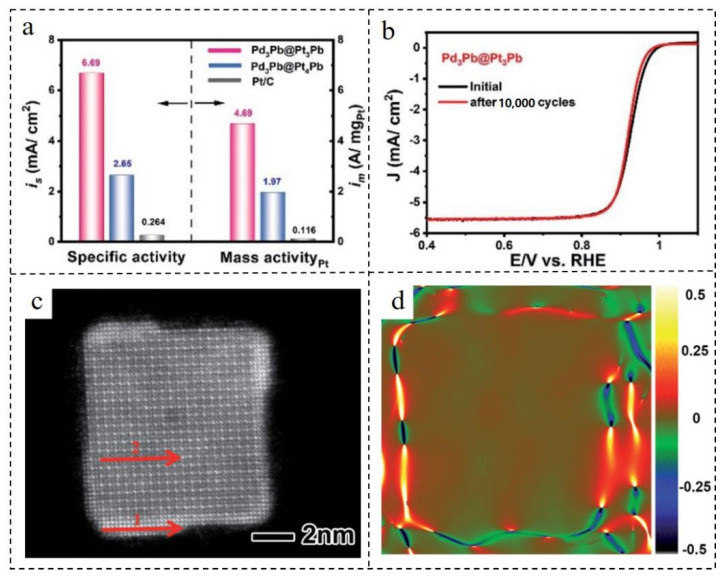
(**a**) Mass activity and specific activity at 0.9 V_RHE_ of Pd_3_Pb@Pt_3_Pb, Pd_3_Pb@Pt_4_Pb and Pt/C. (**b**) LSV polarization curves (100 mV·s^−1^) of the Pd_3_Pb@Pt_3_Pb/C nanocubes before and after 10,000 cycles. (**c**) Aberration-corrected HAADF-STEM image of the Pd_3_-Pb@Pt_3_Pb nanocubes. (**d**) Mapping image of the ε_xx_ distribution of (**c**). Reproduced with permission from [106]. Copyright © 2022, Royal Society of Chemistry.

As an expensive metal element, Pt greatly increases the cost of the catalyst which reduces the performance-to-price ratio of catalytic efficiency. Therefore, a kind of Pd_3_Pb@Pt_3_Pb nanocube with an ultra-thin shell based on the ligand effect was designed [106]. Excellent mass activity (4.69 A·mgPt^−1^) and specific activity (6.69 mA·cm^−2^) of Pd_3_Pb@Pt_3_Pb NPs were obtained in the 0.1 M KOH electrolyte, which were 40.4 and 25.3 times higher than those of commercial Pt/C, respectively. Additionally, these core–shell nanocubes had high stability, losing only 9.3% of their mass activity after 10,000 cycles, while the activity loss of commercial Pt/C was 59.9%. Through the measurement of strain distribution by geometrical phase analysis (GPA) and the characterization of HAADF-STEM, it could be seen that the compression amount of the cube shell and core was 0.96% and 0.61%, respectively (Figure 10). However, according to previous studies, a 0.6% compression strain could only bring about a 4% increase in activity [107,108], which did not match with the practical results. Combined with the calculation and analysis of GPA and density functional theory (DFT) theory, it was found that the Pd_3_Pb@Pt_3_Pb nanocubes with ultra-thin shells were less affected by the strain effect in the process of ORR. The above results show that the ligand effect plays a key part in improving the ORR activity.

## 4. Conclusion and Prospective

At present, ORR catalysts are an important factor affecting the application of fuel cells. It not only determines the energy conversion efficiency and service life of the fuel cell, but is also closely related to the cost of the fuel cell. Core–shell structural noble metal-based catalysts have outstanding comprehensive performances among many ORR catalysts. For example, the core–shell structure greatly reduces the consumption of noble metals, which significantly improves the mass activity and specific activity of noble metals (Table 1). Therefore, such catalysts have wide application prospects in future commercial production. Although core–shell nanostructured noble metal-based ORR catalysts have made great progress in recent years, they still present some serious problems: (1) The ORR performance of most core–shell structural noble metal-based catalysts is only based on the data obtained by half-cell tests, which is very different from the actual fuel cell complex environment. In order to realize the commercial production of the catalyst, it is also necessary to test the core–shell nanostructured noble metal-based ORR catalyst in the real battery environment. (2) Some core–shell structural noble metal-based catalysts have high ORR activity and stability. However, the synthesis route is complex, the reaction conditions are extremely harsh, and various tools are required in the reaction process. The synthesis method can only be used for laboratory preparation but not for large-scale production, so it is necessary to design a simple batch preparation method. (3) There are relatively few studies on the ORR mechanism of core–shell structural noble metal-based catalysts, and most of the research results on these mechanisms were only obtained by conducting theoretical calculations and simulations. Studies on in situ detection technology and advanced instruments for catalytic processes (especially the reaction intermediates) can help to better understand the catalytic mechanism of core–shell structural noble metal-based catalysts. It plays a crucial part in the reasonable design of the catalyst structure and performance.

In this review, the latest progress in the field of core–shell structural noble metal-based ORR catalysts was summarized, and the reaction mechanism and performance testing methods of ORR were briefly explored. Then, the factors affecting the performance of core–shell nanostructured noble metal-based ORR catalyst were discussed, including the synthesis method, temperature, kinds of doping elements and the number of shell layers. The main results are as follows: (1) The preparation methods of core–shell structural noble metal-based catalysts mainly include the electrodeposition method, wet chemical method and electrochemical dealloying method. Although the traditional electrodeposition method can be used to obtain a thin and uniform shell and greatly reduce the use of noble metals, it requires high reaction conditions and instruments. So, the traditional electrodeposition method is not suitable for large-scale production. It is quite meaningful to study ways to reduce the reaction conditions and instrument requirements when using this kind of preparation method. The simple method of chemical reduction is a one-pot reduction, and the core–shell structural noble metal-based catalysts obtained by this method exhibit good activity and stability in ORR. In addition, the direct replacement method and sonochemical method can be used to synthesize core–shell structural catalysts directly in one step. These simple one-step synthesis methods promote the application of core–shell nanostructured noble metal-based ORR catalysts in industrial production. (2) The effect of temperature on the activity of ORR catalysts concerns two main areas. Firstly, the ORR activity and stability of the catalyst are different at different test temperatures. However, there is a great difference in the temperature between the half-cell test and the actual fuel cell working environment. It often leads to the catalyst being unable to achieve the predicted effect in practical application. It can be seen that it is of great significance to understand the variation in the ORR performance and test temperature of core–shell structural noble metal-based catalysts, which will help to maximize the performance of catalysts in practical applications. Secondly, the annealing temperature in the preparation process also has a great influence on the structure and performance of catalysts. When the annealing temperature is higher, the core is mixed with the atoms of the shell. Additionally, when the annealing temperature is lower, the shell is incomplete. By referring to the acceptable annealing temperature of the catalyst, we can analyze whether the catalyst can use the dispersion technology of a large amount of heat production and the assembly and manufacturing technology of a membrane electrode which requires hot pressing. (3) As the most expensive part of the catalyst, noble metals are the greatest obstacle to the commercialization of fuel cells. Therefore, it is necessary to improve the utilization of noble metals in core–shell catalysts in some ways so as to effectively reduce commercial costs. The compression and expansion of the core and shell are controlled by doping impurity atoms into the core and shell, which leads to the strain effect and subsequently changes the surface properties of the shell. It can greatly improve the ORR activity of the catalyst without increasing the dosage of noble metals. The incorporation of transition metal elements such as Fe, Co and Ni can significantly improve the ORR activity of noble metal-based core–shell catalysts. Additionally, this method can help to greatly reduce the dosage of noble metals. However, transition metal elements are easy to dissolve. Such a technique will affect the stability and durability of the catalyst. Fortunately, there are many ways to inhibit the leaching of these elements. So, low-priced transition metals have broad applications in noble metal-based core–shell catalysts. When the shell is thin enough, the strain effect will be more pronounced, but the ligand effect plays a dominant role in the performances of core–shell nanostructured noble metal-based ORR catalysts. It is worth noting that the strain effect and ligand effect sometimes weaken each other and affect the ORR performances. Therefore, choosing the appropriate shell layer number to allow the strain effect and ligand effect to cooperate is also an important consideration in the design of catalysts.

To summarise, as a new generation of electrocatalysts, core–shell structural noble metal-based nanomaterials provide a solid and stable platform for green energy and resource conservation. Moreover, they present great potential in the field of fuel cells. In the near future, core–shell structural noble metal-based catalysts are expected to become one of the main commercial ORR catalysts, which will greatly promote the application progress of fuel cells.

## Figures and Tables

**Table 1:** Summary of ORR activities of core–shell catalysts (measured at 0.9 V).

Core-Shell Materials	Electrolyte	Mass Activity [A·mg_Pt_^−1^]	Specific Activity [mA·cm^−2^]	Refs
Pt@Pd_2_Co/C NPs	0.1 M HClO_4_	0.72	0.5	[29]
Pd_3_Fe@Pt/C NPs	0.1 M HClO_4_	1.14	0.88	[62]
^CG^CuPd/C NPs	0.1 M HClO_4_	2.624	1.11	[63]
PtNi@Pt_D_/G NPs	0.1 M HClO_4_	0.061	0.098	[75]
PtCo@Pt NPs	0.1 M HClO_4_	2.82	9.16	[89]
PtP_1.4_@Pt/C NPs	0.1 M HClO_4_	0.31	0.62	[90]
Pd@PtNiCu NPs	0.1 M HClO_4_	1.79	5.4	[98]
Pd@PtIr NPs	0.1 M HClO_4_	1.88	1.27	[99]
FePt@PtBi NPs	0.1 M HClO_4_	0.96	2.06	[102]
Pd_3_Pb@Pt_3_Pb NPs	0.1 M KOH	4.69	6.69	[106]

## Data Availability

Not applicable.
